# An Account of a Case, in Which a Part of the Femoral Artery Was Dilated in Consequence of Its Being Laid Bare by a Wound, and Which Was Successfully Treated by Obliterating the Cavity of the Artery, at That Part, by Compression

**Published:** 1787

**Authors:** Robert Kinglake

**Affiliations:** Surgeon at Chipping Norton, in Oxfordshire


IV. *An Account of a Case, in which a Part of the Femoral Artery was dilated in consequence of its being laid bare by a Wound, and which was successfully treated by obliterating the Cavity of the Artery, at that Part, by Compression. Communicated in a Letter to Dr.* Simmons *by Mr.* Robert Kinglake, *Surgeon at Chipping Norton, in Oxfordshire.*

RICHARD Rooke, of Barton, in Warwickshire, aged thirty years, and of a robust constitution, was goaded in the thigh,

 about about four months ago, by a bullock. The wound was a lacerated one, and immediately oppofite the middle part of the femoral artery, which very narrowly efcaped divifion. This circumftance of the extreme proximity of the artery to the wound conftitutes the groundwork of what appears in this cafe to merit obfervation.

On my firft infpecting the wound, which was in a few hours after the accident had occurred, I found it filled with coagulated blood, vifibly moving with the pulfations of the fubjacent artery. From hence conceiving the perilous vicinity the artery held with the wound, I apprehended very dangerous confequences. I began, however, to encounter the difficulties by a copious blood-letting, and by well evacuating the bowels; a moderate compreffion was alfo made on the artery, juft below where the profunda is fent off, with a view to diminifh the impulfe of the circulation on the part of the artery connected with the accident, and to afford an opportunity for an increafed quantity of blood to pafs through the collateral branches: but notwithftanding thefe precautions, the part of the artery at the wound, from being deprived of an equal and ufual resistance from the superincumbent integuments, in twenty-four hours was dilated beyond the edges of the wound, which it completely filled up. On pressing the dilatation with my finger, the propulsive force of the heart felt incredibly strong, and required a forcible and steady pressure to resist the impulse. In this precarious state of circumstances, it seemed difficult to determine what course was most eligible; whether to remove the limb for a certain preservation of life, or, for the chance of preserving the limb, to involve the case in all the difficulties resulting from an intercepted and diverted circulation.

After no little hesitation, and finding the patient decidedly averse to amputation, I resolved on making a compression on the dilated artery that would approximate the sides of the vessel at that part, so as to induce an union, and consequent destruction of its continuity. This I was farther encouraged to attempt, from conceiving that the probably inflamed state of the arterial coats, in consequence of the accident, increased under the irritation of the necessary pressure, might insure a coalescence on the principle of adhesive inflammation. In conformity to this idea, I made a compression with an oblong button tourniquet, ſo applied as to make particular and concentered preſſure. The dilatation yielded to the force employed, and remained quiet under the ſuppreſſion. The obſtacle given to the circulation was evinced by an immediate and total loſs of pulſation in the ham. To co-operate in the intention of cure, I made a gentle preſſure on the artery, from the part where it was dilated, nearly as high up as where the profunda goes off.

The effects of obſtructed circulation now began to appear in their uſual terrific form. The part of the thigh above the compreſſion became much ſwollen, inflamed, and extremely painful; feeling, to uſe the patient's own expreſſion, as if the thigh was rending aſunder. The part of the extremity below the compreſſion ſuffered a diminution of its natural heat, with a torpid feel, and was ſoon loaded with œdematous tumefaction. The ſyſtem, in general, alſo partook of the irritation, the functions of the body becoming deranged, and head-ach, bleeding at the noſe, frequent ſickneſs, and occaſional vomiting, being excited.

After two days ſcarcely unvaried continuance of this deplorable ſituation, the pulſe became palpable in the ham, and a ſenſation of glowing warmth was now felt diffufing through the inferior part of the extremity; the fwelling above the compreffion, together with the preternatural heat and pain, began fenfibly to decreafe; and the edges of the wound appeared tumid and digefting. This was on the third day from the application of the compreffion, which I judged to be too early a period either for the flackening or removal of the comprefs. I, therefore, allowed it to remain on for five days longer, during which time every thing continued progreffively in a favourable train, without any formidable interruption.

On removing the comprefs, incarnation was obfervable in the wound, without the fmalleft appearance of an arterial tube. For fecurity, a comprefs, moderately tight, was continued for a month, when the wound was clofed with an indented cicatrix. The patient has ever fince (now nearly three months) followed the daily labour of an hufbandman, without any other inconveniencies than thofe of a more obtufe feeling in the leg and foot than is natural; an unufual fenfe of cold; and finding that, after long ftanding, the leg and foot become a little œdematous. The fwelling, however, goes entirely down by the morning, after he has lain a few a few hours in bed? But these are obvious effects of a want of arterial vigour in the extremity, and will, I should suppose, be surmounted when the collateral branches are rendered more capaciously pervious.

This case may serve as an admonition to surgeons not to think indiscriminately of the danger of arterial dilatations, but always to connect them with their causes; for certainly a very obvious difference exists, in the degree of hazard, between a dilatation ensuing a recent external accident, and one originating from a loss of power or ossific inaction in the coats of an artery. In the former, the dilatation results from mechanical circumstances, the artery, considered abstractedly, remaining sound; in the latter, it is the consequence of weakness, or altered structure, the extent of which cannot be defined: the mode of treatment, therefore, which may be applicable to the former of these cases, and which, in the instance I have related, was successful, would, in the latter, be of very dubious efficacy, as the artery, if compressed at the dilatation, would, from its deficient power, most probably yield to the additional impulse in another part, and frustrate the cure. This view of the subject clearly explains a dissimilarity fimilarity that at once fhews the propriety of this mode of treatment in the one cafe, and as clearly elucidates the extreme incertitude and probable infufficiency of its employment in the other. Should the event of the cafe I have related tend to enforce an imitation of the practice in fimilar circumftances, and be productive of as happy an effect, the fuccefs will be not lefs creditable to furgery than congenial with the feelings of humanity in fuperfeding the truly-horrid alternative — amputation.

*Chipping Norton,*

Oct. 13th, 1787.

